# Setanaxib (GKT137831) Ameliorates Doxorubicin-Induced Cardiotoxicity by Inhibiting the NOX1/NOX4/Reactive Oxygen Species/MAPK Pathway

**DOI:** 10.3389/fphar.2022.823975

**Published:** 2022-04-04

**Authors:** Hui Zheng, Nannan Xu, Zihao Zhang, Fen Wang, Jie Xiao, Xiaoping Ji

**Affiliations:** ^1^ The Key Laboratory of Cardiovascular Remodeling and Function Research, Chinese Ministry of Education, Chinese National Health Commission and Chinese Academy of Medical Sciences, The State and Shandong Province Joint Key Laboratory of Translational Cardiovascular Medicine, Department of Cardiology, Qilu Hospital, Cheeloo College of Medicine, Shandong University, Jinan, China; ^2^ Department of Infectious Diseases, Qilu Hospital, Cheeloo College of Medicine, Shandong University, Jinan, China; ^3^ Department of Cardiology, Weihai Central Hospital, Weihai, China; ^4^ Department of Critical Care Medicine, Qilu Hospital, Cheeloo College of Medicine, Shandong University, Jinan, China

**Keywords:** doxorubicin, cardiotoxicity, GKT137831, NADPH oxidase, apoptosis

## Abstract

**Background:** Doxorubicin (DOX)-induced cardiotoxicity is a highly concerning issue, and the mechanism by which DOX induces cardiotoxicity is likely to be multifactorial. NADPH oxidase (NOX) is associated with DOX-induced cardiotoxicity. Setanaxib (GKT137831), a preferential direct inhibitor of NOX1 and NOX4, can delay or prevent the progression of many cardiovascular disorders by inhibiting reactive oxygen species (ROS) generation. In this study, we investigated the role of GKT137831 in ameliorating DOX-induced cardiotoxicity and the potential mechanisms of its action.

**Methods and Results:** The mice model of cardiotoxicity induced by DOX was established, and GKT137831 treatment was performed at the same time. Neonatal rat cardiomyocytes (NRCMs) were treated with DOX or GKT137831 for *in vitro* experiments. We found that DOX administration impaired cardiac function *in vivo*, reflected by decreased left ventricular ejection fraction (LVEF) and fractional shortening (FS%). DOX also impaired the viability of NRCMs *in vitro*. In addition, DOX increased the levels of NOX1 and NOX4 expression and ROS production and the cardiomyocyte apoptosis rate, both *in vivo* and *in vitro*. GKT137831 improved cardiac function, as indicated by the increased LVEF and FS%. *In vitro,* GKT137831 improved NRCM viability. It also decreased ROS production and the cardiomyocyte apoptosis rate. Apoptotic indices, such as cleaved PARP (c-PARP), cleaved caspase 3 (CC3) and BAX expression levels, were decreased, and the antiapoptotic index of Bcl-2 expression was increased. DOX markedly activated phosphorylated JNK, ERK and p38 proteins in NRCMs. Specific inhibitors of JNK (SP600125), ERK (PD98059) or p38 (SB203580) inhibited DOX-induced apoptosis of NRCMs. GKT137831 pretreatment inhibited excessive DOX-induced MAPK pathway activation.

**Conclusion:** This study revealed that GKT137831 can alleviate DOX-induced cardiomyocyte apoptosis by inhibiting NOX1/4-driven ROS production. The upregulation of MAPK pathway induced by NOX1/4-derived ROS production may be the potential mechanism of GKT137831 action. GKT137831 may be a potential drug candidate to ameliorate DOX-induced cardiotoxicity.

## Introduction

Doxorubicin (DOX), the most commonly used anthracycline, remains a prominent treatment in numerous types of cancers. However, dose-dependent cardiotoxicity induced by DOX became apparent soon after its widespread use in the 1970s (Von Hoff et al., 1979). The exact mechanism by which DOX induces cardiotoxicity is likely multifactorial, involving direct pathways affected by reactive oxygen species (ROS) generation (Singal and Iliskovic, 1998) and topoisomerase 2β (Zhang et al., 2012), as well as other indirect pathways.

NADPH oxidases (NOXs) constitute a group of plasma membrane-associated enzymes with the sole function of producing ROS (Altenhofer et al., 2012). The family has seven members, NOX1-5 and the dual oxidase DUOX1-2. These isoforms differ in their regulation, tissue and cellular distribution, subcellular localization and the ROS type that they produce (Elbatreek et al., 2021). NOX-derived ROS are essential modulators of signal transduction pathways that control key physiological activities (Manea et al., 2015). However, excessive and sustained release of NOX-derived ROS may play pathological roles in various diseases, including atherosclerosis (Sheehan et al., 2011; Douglas et al., 2012), hypertension (Touyz et al., 2019), diabetic kidney disease (Jha et al., 2016) and lung fibrosis (Louzada et al., 2021). Furthermore, NOX has been proposed to be involved in another potential enzymatic system leading to ROS production after DOX treatment, and NOX-derived ROS may be involved in the pathological development of DOX-induced cardiotoxicity (Zeng C. et al., 2020; Cheng et al., 2020).

Setanaxib (GKT137831) is a specific dual NOX1 and NOX4 inhibitor. Recent studies have shown that GKT137831 plays a protective role in many cardiovascular disorders. For instance, GKT137831 attenuated the development of diabetes-associated atherosclerosis (Gray et al., 2013) and prevented hypertensive cardiac remodeling and hypertrophy (Zhao et al., 2015; Zeng et al., 2019). However, studies investigating the role of GKT137831 in DOX-induced cardiotoxicity are rare.

Considering the important role of NOX in DOX-induced cardiotoxicity, we hypothesize that GKT137831 may reduce NOX1/NOX4-derived ROS production, thereby reducing ROS-induced apoptosis, and the potential mechanisms of GKT137831 action is discussed in this study.

## Materials and Methods

### Reagents and Antibodies

DOX (MB1087) was obtained from Dalian Meilun Biotechnology Co., Ltd., China. Setanaxib (GKT137831), sodium carboxymethyl cellulose (CMC-Na; S6703), acetylcysteine (NAC; S1623), SP600125 (S1460), PD98059 (S1177), and SB203580 (S1076) were obtained from Selleck Chemicals (Houston, TX, United States). An *In Situ* Cell Death Detection kit, TMR red, for use at room temperature (RT) (cat. no. 12156792910) was obtained from Roche Diagnostics (Mannheim, Germany). A lactate dehydrogenase (LDH) assay kit (C0016), JC-1 kit (C2006), and ROS assay kit (S0033S) were obtained from Beyotime (Shanghai, China). A frozen section reactive oxygen detection kit (BB-470513) was obtained from Shanghai Beibo Biotechnology Co., Ltd., China.

Antibodies against NOX4 were purchased from Invitrogen (PA5-53304, CA, United States), and Abcam (ab133303, Cambridge, United Kingdom). An antibody against NOX1 (PA5-103220) was purchased from Invitrogen. An antibody against cardiac troponin I (66376-1-Ig) was purchased from Proteintech (Wuhan, China). Antibodies against PARP (#9542), cleaved caspase 3 (#9661), JNK (#9252), p-p44/42 MAPK (Erk1/2) (#4370), p44/42 MAPK (Erk1/2) (#4695), p-p38 MAPK (#4511), p38 MAPK (#8690), and β-actin (#4970) were purchased from Cell Signaling Technology (Shanghai, China). Antibodies against Bcl-2 (ab182858), Bax (ab32503), 4-Hydroxynonenal (4-HNE, ab46545), and p-JNK (ab76572) were purchased from Abcam.

### Animal Experiments

Eight-week-old male C57BL/6J mice were obtained from ViewSolid Biotech (Beijing, China). After 1 week of adaptive feeding, the mice were randomly assigned to four groups: a control, control + GKT137831, DOX, and DOX + GKT137831 group, *n* = 10, 10, 20, 20 per group, respectively. To establish the DOX and DOX + GKT137831 groups, mice were injected with a cumulative dose of 20 mg/kg DOX [5 mg/kg Intraperitoneal (i.p.) injection at 0, 7, 14, and 21 days], while an equivalent volume of saline was administered by i.p. injection to the control and control + GKT137831 groups. After the first injection, the control + GKT137831 and DOX + GKT137831 group mice were treated with GKT137831 (60 mg/kg/d) by gavage, and an equivalent volume of 0.5% CMC-Na was administered to the control and DOX group mice by gavage. The mice were euthanized 6 weeks after the first injection. All animal experimental protocols complied with the Animal Management Rules of the Chinese Ministry of Health (document no. 55, 2001) and conformed to National Institutes of Health (NIH) guidelines (the Guide for the Care and Use of Laboratory Animals; NIH Publication No. 85-23, revised 1996).

### Echocardiography

Transthoracic echocardiography was performed using a VisualSonics Vevo 2100 system equipped with an MS400 transducer (Visual Sonics). Anesthesia (5% isoflurane) was administered, and the mice remained under general anesthesia with continuous administration of 2% isoflurane during echocardiogram acquisition. Indices of systolic function were obtained from long-axis M-mode scans. The parameters collected included left ventricular ejection fraction (LVEF), fractional shortening (FS%), end-systolic interventricular septal wall thickness (IVSS), end-diastolic interventricular septal wall thickness (IVSD), left ventricular end-systolic diameter (LVESD), left ventricular end-diastolic diameter (LVEDD), left ventricular end-systolic posterior wall (LVPWs), and left ventricular end-diastolic posterior wall (LVPWd).

### Histological Analysis

Heart tissues were harvested, washed, fixed immediately in 4% paraformaldehyde and embedded in paraffin. Five-micrometer sections were collected and subjected to hematoxylin and eosin (HE) and Masson’s trichrome staining. Immunofluorescence (IF) was performed to assess the expression of NOX1 and NOX4 after DOX treatment *in vivo*. To monitor oxidative stress status, frozen heart sections were stained with dihydroethidium (DHE). Transmission electron microscopy (TEM) was used to observe ultrastructural changes.

### Immunofluorescence

After paraffin-embedded mouse heart section rehydration, heat antigen retrieval, 3% H_2_O_2_ treatment, 0.4% Triton X-100 (T8200; Solarbio, Beijing, China) treatment and washing with PBS, slides with the sections were blocked with 5% goat serum (SL038; Solarbio) for 30 min at 37°C, followed by overnight incubation with primary antibodies against NOX1 (1:200 dilution), NOX4 (1:200 dilution), and cTnI (1:200 dilution) at 4°C in a humid chamber. After washing, the slides were incubated with secondary antibodies (1:200 dilution, ZSGB-BIO) for 1 h at 37°C. The slides were covered with a drop of DAPI (Abcam) before being observed with a microscope (Nikon).

### Neonatal Rat Rardiomyocytes Culture and Treatment

Culture of primary NRCMs was prepared as described previously (Li et al., 2014). NRCMs were incubated in high-glucose DMEM (HG-DMEM) supplemented with 8% horse serum, 5% newborn calf serum, penicillin (100 U/ml), streptomycin (100 mg/ml) and 100 μM bromodeoxyuridine. The cells were then diluted and plated in different culture dishes according to the specific experimental requirements. 24 h after plating, the culture medium was changed to HG-DMEM supplemented with 1.6% horse serum, 1% newborn calf serum, penicillin (100 U/ml), streptomycin (100 mg/ml) and 100 μM bromodeoxyuridine for another 48 h. On the fourth day after plating, experiments were initiated. In some experiments, NRCMs were treated with different concentrations of DOX for 24 h or pretreated with GKT137831 (5 μmol/L) for 1 h, NAC (10 mmol/L) for 1 h, SP600125 (10 μmol/L) for 1 h, PD98059 (10 μmol/L) for 1 h, SB203580 (10 μmol/L) for 1 h and then stimulated with DOX (2 μmol/L) for 24 h. Cells were then processed for further examination, such as IF microscopy or Western blotting.

### Cell Injury Evaluation

Cell damage was assessed by determining the level of LDH from the cells with an LDH detection kit according to the manufacturer’s instructions. The absorbance of the samples was measured with a microplate reader at a wavelength of 490 nm.

### Measurement of Reactive Oxygen Species

Evaluation of the level of intracellular ROS was carried out with a reactive oxygen species assay kit. Measurements were based on the manufacturer’s instructions. Cells treated with Rosup (50 μg/ml) for 20 min were used as positive controls.

### Western Blot Analysis

Proteins were separated by 10% or 12% sodium dodecyl sulfate-polyacrylamide gel electrophoresis (SDS-PAGE) and then transferred to PVDF membranes (Millipore, United States). After the membranes were blocked with 5% skimmed milk for 1 h at room temperature, they were incubated with primary antibodies against NOX4 (1:1,000), NOX1 (1:1,000), PARP (1:1,000), Bcl-2 (1:2,000), BAX (1:1,000), cleaved caspase-3 (1:1,000), 4-HNE (1:2,000), p-JNK (1:5,000), JNK (1:1,000), p-ERK (1:1,000), ERK (1:1,000), p-p38 (1:1,000), p38 (1:1,000), and β-actin (1:1,000) overnight at 4°C. The membranes were then washed with TBST, followed by incubation with the appropriate horseradish peroxidase-conjugated secondary antibody (1:5,000 dilution; ZSGB-BIO, Beijing, China) for 1 h at room temperature. Protein bands were visualized using an Amersham Imager 680 electrochemiluminescence instrument (General Electric Company).

### JC-1 Staining

Mitochondrial membrane potential (ΔΨm) was measured with a JC-1 kit based on the manufacturer’s instructions. Briefly, after removing the culture medium, the cells were rinsed with PBS, and 500 µl of fresh medium was added, and then, 500 µl of JC-1 stain was added and incubated for 20 min in a 37°C incubator with 5% CO_2_. Then, the supernatant was removed, and the cells were washed twice with JC-1 stain (1×). Next, we added 1 ml of fresh HG-DMEM. We then observed and photographed the cells with a laser scanning confocal microscope (LSM710; Zeiss).

### TUNEL Staining

Apoptotic cells in the myocardium and the neonatal rat cardiomyocyte (NRCM) were detected by the use of a commercial DNA fragmentation detection kit (*In Situ* Cell Death Detection Kit, TMR red; Roche) followed by the manufacturer’s instructions.

### Statistics Analysis

The results are expressed as the means ± SD. All experiments were independently repeated at least three times. Statistical comparisons between 2 groups were performed *via* Student’s t-test (data with normal distribution and homogeneity of variance) or Mann-Whitney test (data not normally distributed or without homogeneity of variance). Statistical comparisons between multiple groups were carried out by one-way ANOVA followed by LSD test (data with normal distribution and homogeneity of variance) or Kruskal-Wallis test followed by Dunn’s test (data not normally distributed or without homogeneity of variance). Kaplan-Meier survival analysis was completed using the Log-rank (Mantel-Cox) test. The data were analyzed using SPSS 26.0 and GraphPad Prism 8.0 software. *p* < 0.05 was considered to be statistically significant.

## Results

### The Protein Levels of NOX1 and NOX4 Were Increased by Doxorubicin Both *in vivo* and *in vitro*

**FIGURE 1:**
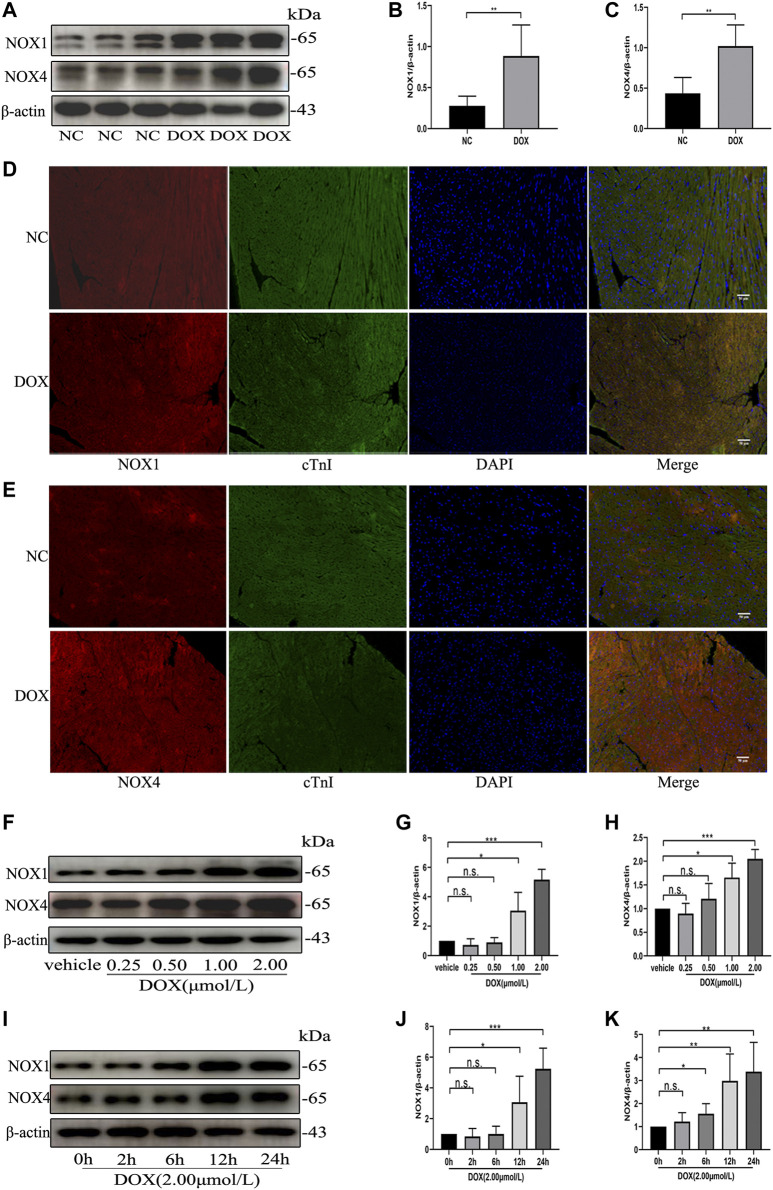
The protein levels of NOX1 and NOX4 were increased by DOX both *in vivo* and *in vitro*. **(A)** Representative western blot analysis of NOX1 and NOX4 in myocardial tissue. **(B,C)** Quantification of NOX1 and NOX4 expression relative to the β-actin level (*n* = 6 per group). **(D,E)** Representative images of NOX1 and NOX4 expression in myocardial tissue detected by the immunofluorescence. The nuclei were stained with DAPI (Scale bar = 50 µm). **(F)** Representative western blot analysis of NOX1 and NOX4 of NRCMs treated with DOX of different concentrations for 24 h **(G,H)** Quantification of NOX1 and NOX4 expression relative to the β-actin level (*n* = 3). **(I)** Representative western blot analysis of NOX 1 and NOX 4 of NRCMs treated with DOX for different time. **(J,K)** Quantification of NOX1 and NOX4 expression relative to the β-actin level (*n* = 4–5). *p < 0.05, **p < 0.01, ***p < 0.001, n. s., not significant. NOX, NADPH oxidase; DOX, doxorubicin; cTnI, Cardiac troponin I.

We first examined the effect of DOX on Nox1 and Nox4 protein expression in the mouse myocardium. As shown in Figures 1A–E, Western blot and immunofluorescence analyses demonstrated that the protein levels of NOX1 and NOX4 were significantly increased in the DOX-treated mouse myocardium, comparing with the control group. The Western blot analysis also demonstrated that the protein expression of NOX1 and NOX4 in the NRCMs was increased in a dose-dependent and time-dependent manner (Figures 1F–K) after DOX exposure. These results indicated that upregulation of NOX1 and NOX4 expression might be involved in DOX-induced cardiotoxicity.

### GKT137831 Alleviated Doxorubicin-Induced Cardiac Dysfunction *in vivo* and Attenuated Diminished Cell Viability *in vitro*

**FIGURE 2:**
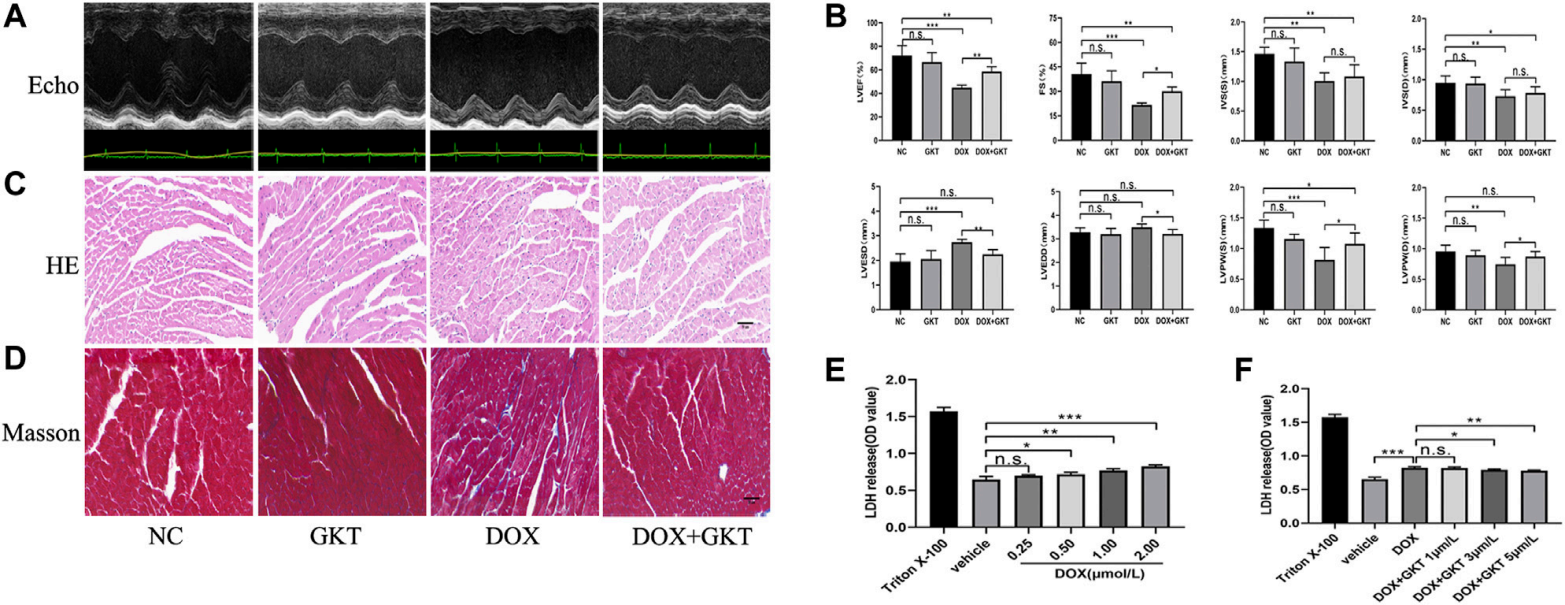
GKT137831 alleviated DOX-induced cardiac dysfunction *in vivo* and attenuated diminished cell viability *in vitro*. **(A)** Representative M-mode echocardiograms. **(B)** Echocardiographic analysis of LVEF, FS, IVSS, IVSD, LVESD, LVEDD, LVPWS, and LVPWD (*n* = 5–6 per group). **(C,D)** Representative images with HE and Masson’s trichrome staining in myocardial tissue (scar bar = 50 µm). **(E)** NRCMs were stimulated with DOX for 24 h and followed by LDH assay to evaluate the cell viability (*n* = 4). **(F)** NRCMs were pretreated with different concentrations of GKT for 1 h before DOX (2 μM) stimulation. The cell viability was evaluated by LDH assay (*n* = 4). *p < 0.05, **p < 0.01, ***p < 0.001, n. s., not significant. Echo, echocardiography; DOX, doxorubicin; GKT, GKT137831; LVEF, left ventricular ejection fraction; FS, fractional shortening; IVSS, end-systolic inter-ventricular septal wall thickness; IVSD, end-diastolic inter-ventricular septal wall thickness; LVESD, left ventricular end-systolic diameter; LVEDD, left ventricular end-diastolic diameter; LVPWS, left ventricular end-systolic posterior wall; LVPWD, left ventricular end-diastolic posterior wall; LDH, lactic dehydrogenase.

After 6 weeks of treatment, all mice in the control group and the control + GKT137831 group survived. In contrast, 55% (11/20) mice survived in the DOX-treated group and 65% (13/20) mice survived in the DOX + GKT137831 group, and there was no significant difference between the two groups (*p* > 0.05, Supplementary Figure S1). As depicted in Figures 2A,B, the graph of echocardiogram data and the statistical results showed that DOX administration decreased the LVEF (EF%), FS (FS%), thickness of IVS, LVPW and increased the LVESD. Though LVEDD increased, which had no significant difference compared with the control group. GKT137831 treatment was highly effective in attenuating DOX-induced LV dilation and worsening of EF% and FS%, although GKT137831 itself exhibited little effect. *In vivo*, HE and Masson’s staining showed that the cardiomyocytes in the DOX-treated group were disorganized, with increased cytoplasmic vacuolization and myocardial fibrosis. Following treatment with GKT137831, DOX-induced cardiomyocyte injury was alleviated, as indicated by attenuated morphological changes and decreased cytoplasmic vacuolization and fibrosis (Figures 2C,D). *In vitro*, we stimulated NRCMs with DOX to determine whether pretreatment with GKT137831 attenuated diminished cell viability by detecting the release of LDH from NRCMs. Increased LDH release is conversely correlated with cell activity. As shown in Figure 2E, DOX stimulation resulted in reduced cell viability in a dose-dependent manner; however, pretreatment with GKT137831 attenuated DOX-induced cardiotoxicity (Figure 2F). These results indicated that GKT137831, a dual NOX1 and NOX4 inhibitor, may play a protective role in DOX-induced cardiotoxicity.

### GKT137831 Ameliorated Oxidative Stress Both *in vivo* and *in vitro*

**FIGURE 3:**
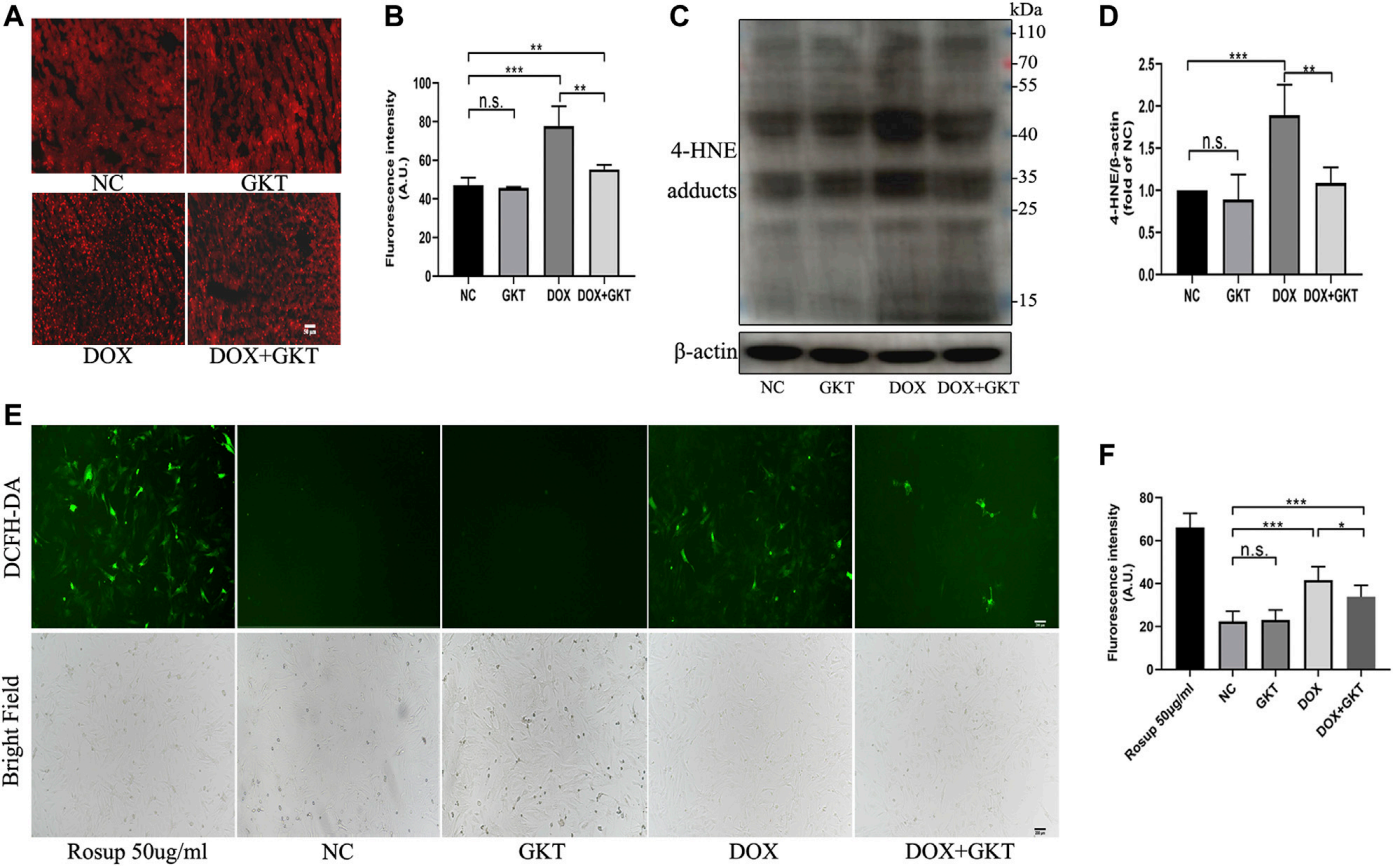
GKT137831 ameliorated oxidative stress both *in vivo* and *in vitro*. **(A,B)** Representative images of myocardial tissue with DHE staining (scar bar = 50 µm) and fluorescence intensity analysis in different groups (*n* = 5 per group). **(C,D)** Representative images of western blotting for 4-HNE and quantifications by densitometric analysis, β-actin was used as a loading control (*n* = 5 per group). **(E,F)** Intracellular ROS generation in NRCMs was detected using DCFH-DA probe then evaluated by fluorescence microscopy (scar bar = 200 µm) and fluorescence intensity analysis. NRCMs were pretreated with GKT (5 μM) for 1 h, and then treated with DOX (2 μM) for 24 h. Rosup was used as a ROS positive control (*n* = 3). *p < 0.05, **p < 0.01, ***p < 0.001, n. s., not significant. Abbreviations: DOX, doxorubicin; GKT, GKT137831; DHE, dihydroethidium: 4-HNE, 4-hydroxynonenal: DCFH-DA, dichlorodihydrofluorescein diacetate; AU, arbitrary unit.

First, the Western blot analysis indicated that GKT137831 treatment could inhibit the protein expression of NOX1 and NOX4 compared with the DOX-treated group both *in vivo* and *in vitro* (Supplementary Figure S2). Oxidative stress is suggested to be the main cause of DOX-induced cardiotoxicity, thus we detected ROS levels both *in vivo* and *in vitro*. *In vivo*, compared with the control mice, the DHE fluorescence intensity of DOX treated mice was significantly enhanced (Figures 3A,B). Western blot-determined level of 4-HNE was also remarkably increased in the hearts of DOX-treated mice (Figures 3C,D). These effects were ameliorated by GKT137831 treatment. *In vitro*, a DCFH-DA fluorescence probe was used to measure intracellular ROS in the different groups. As shown in Figures 3E,F, GKT137831 pretreatment ameliorated DOX-induced ROS production, as measured by the green fluorescence intensity of DCFH-DA. All these results indicated that GKT137831 ameliorates NOX1/4-derived ROS both *in vivo* and *in vitro*.

### GKT137831 Alleviated Doxorubicin-Induced Mitochondrial Damage Both *in vivo* and *in vitro*

**FIGURE 4:**
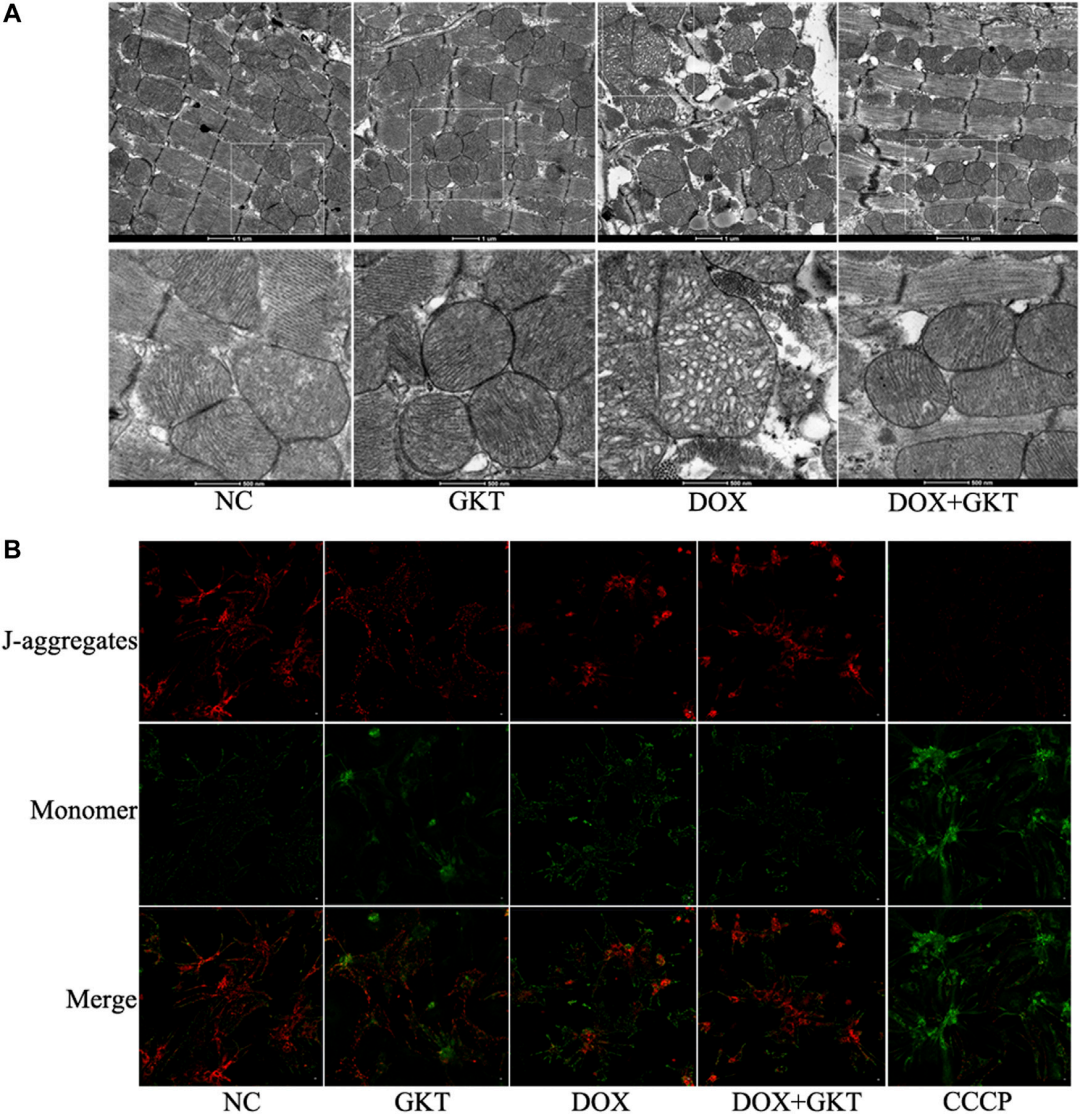
GKT137831 alleviated DOX-induced mitochondrial damage both *in vivo* and *in vitro*. **(A)** Representative transmission electron microscopy image of cardiac mitochondrial ultrastructure. **(B)** Mitochondrial membrane potential measured with JC-1 staining (scale bar = 50 µm). CCCP was used as a positive control. DOX, doxorubicin; GKT, GKT137831; CCCP, carbonyl cyanide m-chlorophenylhydrazone.

Considering that mitochondria are the major subcellular targets of DOX, we performed TEM to examine mitochondrial morphology *in vivo*. We found that DOX induced abnormal changes in mitochondrial structure, including irregular arrangement, swelling, vacuolated and disrupted cristae in the mouse heart. The effects were alleviated by GKT137831 treatment (Figure 4A). *In vitro*, JC-1 staining indicated that Δψm disruption triggered by DOX was partially restored by pretreatment with GKT137831 (Figure 4B). These results indicated that GKT137831 may alleviate DOX-induced mitochondrial damage.

### GKT137831 Reduced Doxorubicin-Induced Cardiomyocyte Apoptosis *in vivo*

**FIGURE 5:**
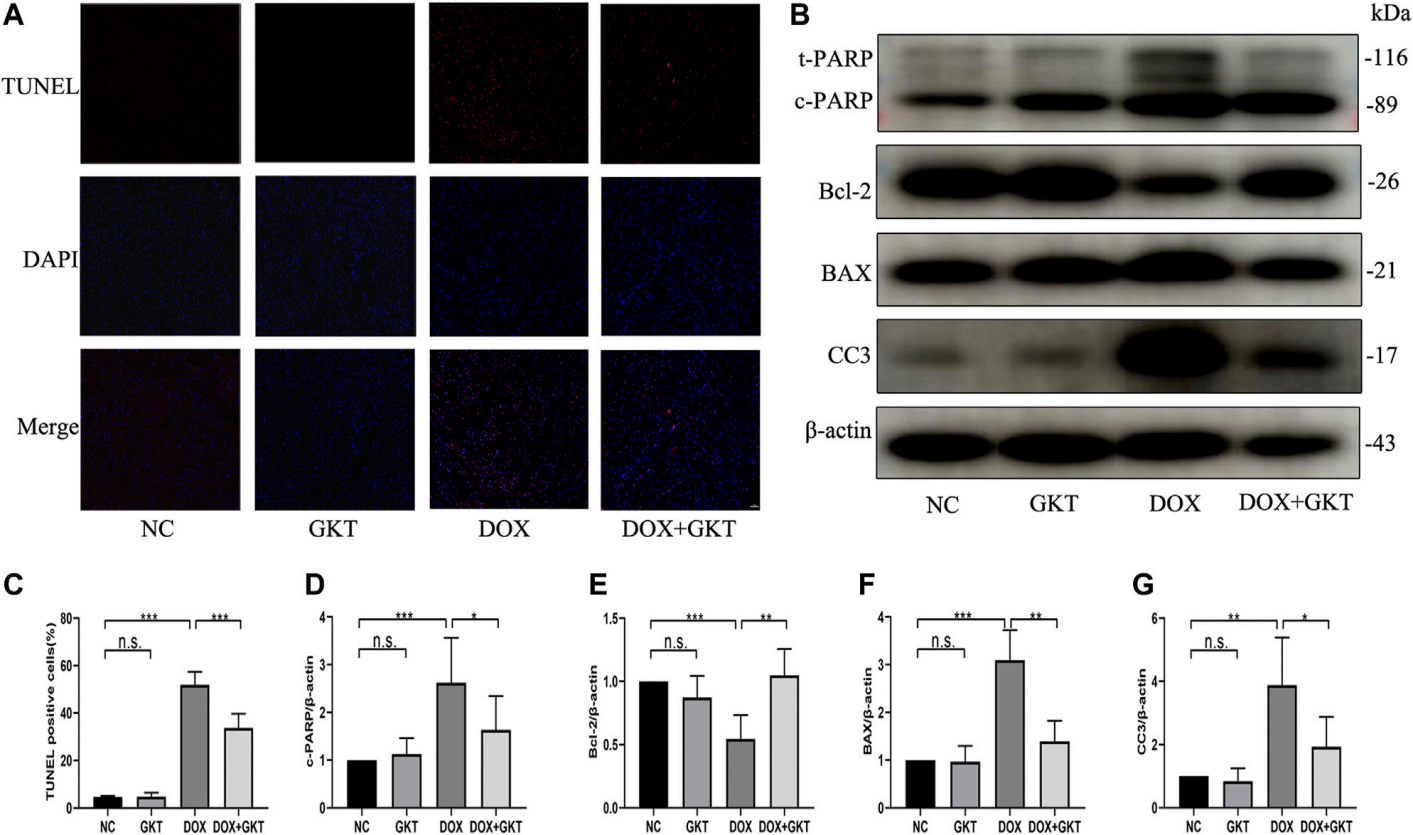
GKT137831 reduced DOX-induced cardiomyocyte apoptosis *in vivo*. **(A,C)** Representative images of TUNEL staining in myocardial tissue (scale bar = 50 μm) and related quantification of TUNEL staining (*n* = 5 per group). **(B)** Representative western blot analysis of c-PARP, Bcl-2, BAX, and CC3 in myocardial tissue, and quantifications by densitometric analysis **(D,E,F,G)**, β-actin was used as a loading control (*n* = 5–7 per group). *p < 0.05, **p < 0.01, ***p < 0.001, n. s., not significant. DOX, doxorubicin; GKT, GKT137831; c-PARP, cleaved PARP; CC3, cleaved caspase 3.

Excess levels of ROS can lead to activation of cell death processes such as apoptosis (Redza-Dutordoir and Averill-Bates, 2016). The proportion of TUNEL-positive cells was significantly increased in DOX-treated mouse hearts. In contrast, fewer TUNEL-positive cells were observed in the GKT137831-treated mice subjected to DOX. GKT137831 treatment itself did not affect the TUNEL-positive cells in the absence of DOX treatment (Figures 5A,C). A Western blot analysis demonstrated that the expression of cleaved PARP, BAX and cleaved caspase three was significantly increased, but the expression level of Bcl-2 was downregulated in DOX-treated hearts compared with control hearts, suggesting activation of the apoptotic pathway, and these effects were suppressed by GKT137831 treatment (Figures 5B,D–G). These results indicated that GKT137831 may ameliorate DOX-induced cardiomyocyte apoptosis *in vivo* by suppressing NOX1/4-derived ROS production.

### GKT137831 Reduced Doxorubicin-Induced Apoptosis of Neonatal Rat Cardiomyocytes

**FIGURE 6:**
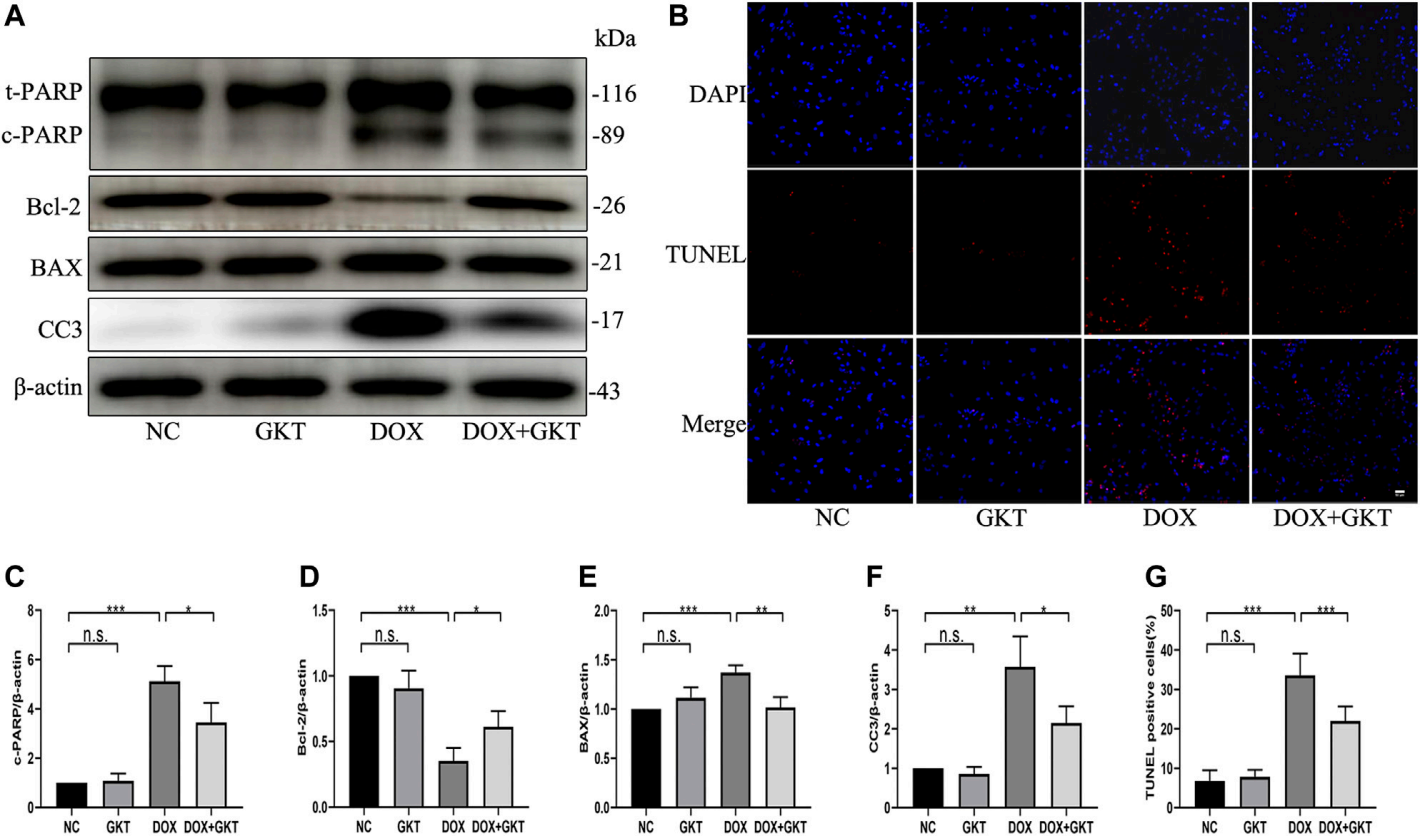
GKT137831 reduced DOX-induced apoptosis of NRCMs. **(A)** Representative western blot analysis of c-PARP, Bcl-2, BAX, and CC3 and quantifications by densitometric analysis **(C,D,E,F)**, β-actin was used as a loading control (*n* = 3). **(B,G)** Representative images of TUNEL staining in NRCMs (scale bar = 50 μm) and related quantification of TUNEL staining (*n* = 3). NRCMs were pretreated with GKT (5 μM) for 1 h, and then treated with DOX (2 μM) for 24 h. *p < 0.05, **p < 0.01, ***p < 0.001, n. s., not significant. DOX, doxorubicin; GKT, GKT137831; c-PARP, cleaved PARP; CC3, cleaved caspase 3.

Next, we stimulated NRCMs with DOX to determine whether GKT137831 pretreatment reduced DOX-induced apoptosis *in vitro*. As shown in Figures 6A,C–F, pretreatment with GKT137831 attenuated DOX-induced apoptosis, which was confirmed by Western blot analysis of cleaved PARP, Bcl-2, BAX and cleaved caspase three expression. In addition, TUNEL staining revealed that pretreatment with GKT137831 significantly decreased the percentage of TUNEL-positive cells (Figures 6B,G). These results indicated that GKT137831 may ameliorate DOX-induced apoptosis in NRCMs by suppressing NOX1/4-derived ROS.

### GKT137831 Ameliorated Doxorubicin-Induced Apoptosis by Inhibiting the MAPK Pathway

**FIGURE 7:**
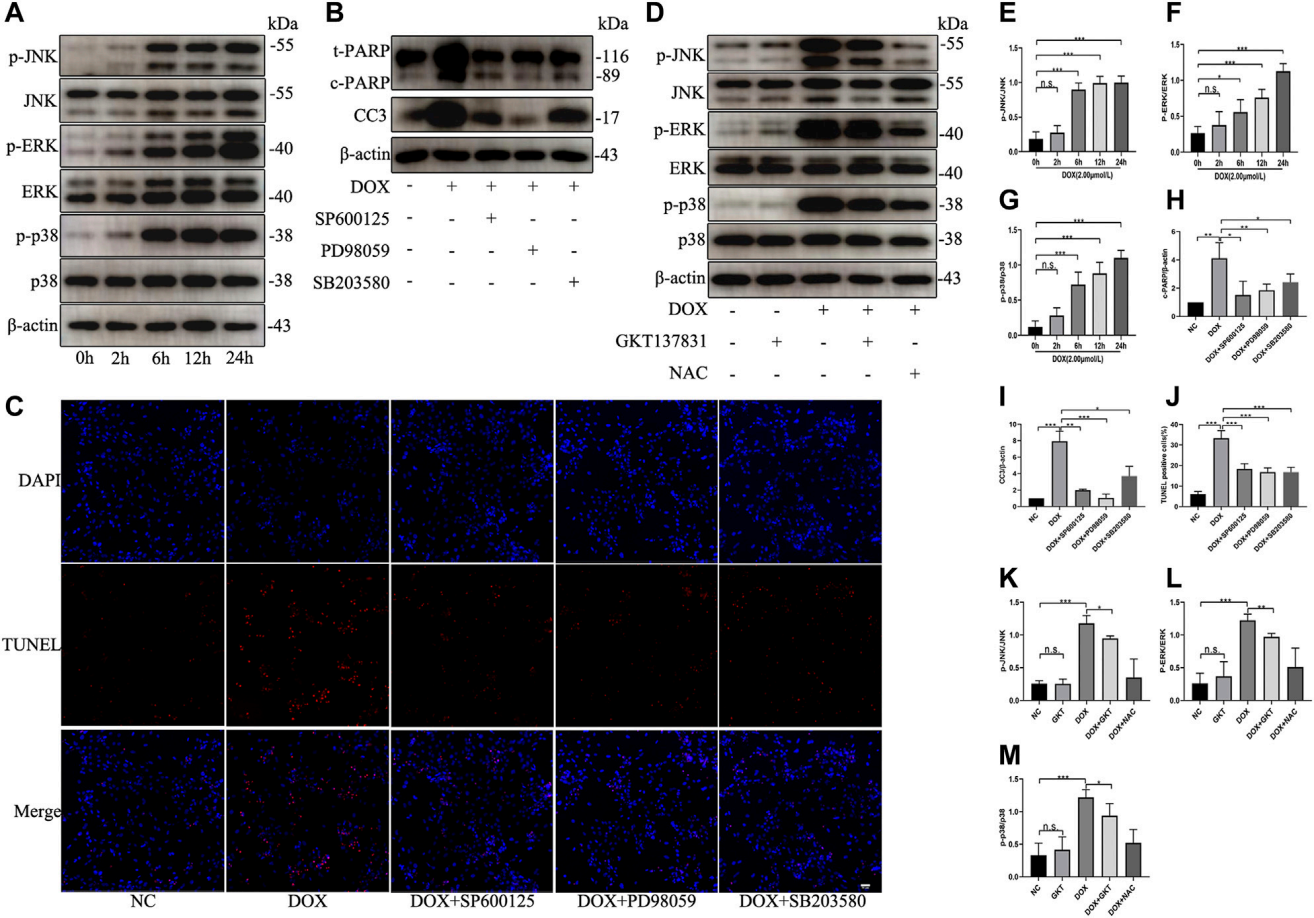
GKT137831 ameliorated DOX-induced apoptosis by inhibiting the MAPK pathway. **(A)** Time-dependent effects of DOX on activation of JNK, ERK, and p38 MAPK were evaluated by Western blot. β-actin was used as a loading control. Relative protein expression levels of p-JNK/JNK **(E)**, p-ERK/ERK **(F)**, and p-p38/p38 **(G)** were quantified (*n* = 3–4). **(B)** NRCMs were pretreated with SP600125 (JNK inhibitor) or PD098059 (ERK1/2 inhibitor) or with SB203580 (p38 inhibitor) for 1 h and then treated with DOX for 24 h **(H,I)** The protein expressions of c-PARP and CC3 were determined by Western blot and quantifications by densitometric analysis (*n* = 3–4). **(C,J)** Representative images of TUNEL staining in NRCMs (scale bar = 50 μm) and related quantification of TUNEL staining (*n* = 3). **(D)** NRCMs pretreated with GKT or NAC for 1 h, and then treated with DOX for 24 h **(K,L,M)** The phosphorylation levels of JNK, ERK, and p38 were evaluated by Western blot, and quantifications by densitometric analysis (*n* = 3–4). *p < 0.05, **p < 0.01, ***p < 0.001, n. s., not significant. DOX, doxorubicin; GKT, GKT137831; NAC, acetylcysteine; c-PARP, cleaved PARP; CC3, cleaved caspase 3.

To test the effect of DOX on the activation of MAPK pathway, NRCMs were treated with DOX for the indicated times. As shown in Figures 7A,E–G, DOX enhanced the phosphorylation and activation of JNK, ERK, and p38 in a time-dependent manner; however, DOX treatment did not induce significant changes in the total levels of JNK, ERK or p38. When we used specific inhibitors against JNK (SP600125), ERK (PD98059) or p38 (SB203580), the levels of the apoptosis indicators cleaved PARP and cleaved caspase three were decreased (Figures 7B,H,I). As shown in Figures 7C,J, the proportion of TUNEL-positive NRCMs was markedly decreased. After pretreatment with GKT137831, the phosphorylation and activation of JNK, ERK, and p38 were decreased in DOX-treated NRCMs. To confirm that ROS can trigger MAPK pathway, NAC was used as the positive control (Figures 7D,K–M). These results revealed that NOX1/4-derived ROS participate in the activation of MAPK pathway and that GKT137831 ameliorates DOX-induced apoptosis by inhibiting MAPK pathway activation to a certain extent.

## Discussion

DOX, the most commonly used anthracycline, plays a prominent role in many cancer treatments. Despite its potency, the clinical application of DOX is limited by its cumulative and dose-related cardiotoxicity. The exact mechanism of DOX-induced cardiotoxicity is multifactorial, and the most widely accepted hypothesis suggests that DOX is related to ROS production (Singal and Iliskovic, 1998). In the myocardium, mitochondria are the major subcellular target of DOX, and its inner membrane consists of cardiolipin, which shows a strong affinity for DOX (Goormaghtigh et al., 1986; Goormaghtigh et al., 1990). Redox cycling of DOX-derived quinone–semiquinone by the NADH dehydrogenase (complex I) of the mitochondrial electron transport chain and the Haber-Weiss reaction without enzymatic involvement are considered to be the major sources of ROS after DOX challenge (Davies and Doroshow, 1986; Berthiaume and Wallace, 2007). However, neither antioxidant nor iron chelation can completely prevent DOX-induced cardiotoxicity (McGowan et al., 2017), suggesting the complexity of the oxidative stress induced.

NADPH oxidase (NOX) is a multicomponent enzyme complex that consists of the membrane-bound subunits gp91^phox^ and p22^phox^, the cytosolic regulatory subunits p47^phox^ and p67^phox^, and the small GTP-binding protein Rac1 (Sumimoto, 2008). It has been proposed that another potential enzymatic system leads to ROS production after DOX treatment. The NOX complex plays an important role in enhancing superoxide production caused by the chemical interaction of DOX and NADPH (Deng et al., 2007). DOX can promote NOX activation, and a series of studies have demonstrated that NOX-derived ROS are implicated in DOX-induced cardiac apoptosis (Gilleron et al., 2009; Zhao et al., 2010). For example, gp91phox-knockout (Deng et al., 2007) or NOX2-deficient mice are resistant to DOX-induced cardiotoxicity (Wojnowski et al., 2005; Zhao et al., 2010; McLaughlin et al., 2017); Rac1 genetic deletion in cardiomyocytes and its inhibitor NSC23766 showed protection against DOX-induced cardiotoxicity (Ma et al., 2013). Therefore, targeting NOXs may be an effective way to reduce DOX-induced cardiotoxicity.

GKT137831 is a preferential direct inhibitor of NOX1 and NOX4 (Elbatreek et al., 2021). It has good pharmacokinetic properties and bioavailability with one or two daily doses administered to rodents or patients (Gorin et al., 2015; Gage and Thippeswamy, 2021). It is also well tolerated and delays or prevents the progression of a range of cardiovascular disorders. For example, GKT137831 attenuated plaque formation by inhibiting ROS generation and reducing the adhesion of inflammatory cells to the vascular wall (Gray et al., 2013); it also markedly attenuated cardiac remodeling by decreasing ROS levels (Zhao et al., 2015) and attenuated hypertensive cardiac hypertrophy by suppressing cardiac inflammation and activating Akt and ERK1/2 (Zeng SY. et al., 2020). It has also been recently tested in clinical trials focused on certain noncardiovascular disorders; for example, a phase II clinical trial with type 2 diabetes nephropathy patients (NCT02010242) and a phase II clinical trial with primary biliary cholangitis patients (NCT03226067) showed GKT137831 efficacy at several primary or secondary endpoints.

Our results showed that DOX increased the levels of NOX1 and NOX4 expression, which was accompanied by elevated ROS production both *in vivo* and *in vitro*. Moreover, DOX triggered apoptosis by increasing the levels of cleaved PARP, cleaved caspase 3, and BAX and reducing the Bcl-2 level. However, GKT137831 treatment decreased ROS generation and attenuated apoptosis. These results indicated that GKT137831 treatment can inhibit NOX1/4-derived ROS production to relieve DOX-induced cardiomyocyte apoptosis.

Then, we further explored the potential mechanism by which GKT137831 reduced DOX-induced myocardium apoptosis. ROS can trigger apoptosis *via* multiple mechanisms, and MAPK pathway is established redox-sensitive mediators involved in the modulation of apoptosis (Son et al., 2011; Kumar et al., 2020). In the present study, we found that DOX exposure activated MAPK pathway in NRCMs. Specific inhibitors against JNK (SP600125), ERK (PD98059) or p38 (SB203580) may abolish DOX-induced apoptosis of NRCMs, and the levels of the apoptosis indicators cleaved PARP and cleaved caspase three were decreased. GKT137831 pretreatment inhibited excessive DOX-induced MAPK pathway activation. These results indicated that GKT137831 attenuates cardiac apoptosis possibly through modulation of MAPK signaling pathway, although the precise mechanism remains to be determined.

In this study, GKT137831 was administered as a protective agent immediately after DOX exposure, and further investigations are required to address whether delayed GKT137831 intervention can attenuate established cardiomyopathy. Additional studies are also needed to determine whether GKT137831 protects against DOX-associated cardiotoxicity without compromising its antitumor effects.

## Conclusion

Our studies demonstrated that GKT137831 can inhibit Nox1/4-derived ROS production to relieve DOX-induced cardiomyocyte apoptosis. Mechanistically, GKT137831inhibits the excessive activation of MAPK pathway induced by Nox1/4-derived ROS. Thus, GKT137831 may be a potential drug candidate to ameliorate DOX-induced cardiotoxicity.

## Data Availability

The original contributions presented in the study are included in the article/Supplementary Material, further inquiries can be directed to the corresponding authors.
