# Supplementary material for: Setanaxib (GKT137831) Ameliorates Doxorubicin-Induced Cardiotoxicity by Inhibiting the NOX1/NOX4/Reactive Oxygen Species/MAPK Pathway
Source: Front Pharmacol. 2022 Apr 4;13:823975. doi: 10.3389/fphar.2022.823975 (PMC9014097; doi:10.3389/fphar.2022.823975)
Supplement: Supplementary file 1 [file DataSheet1.DOCX]

Supplementary Material


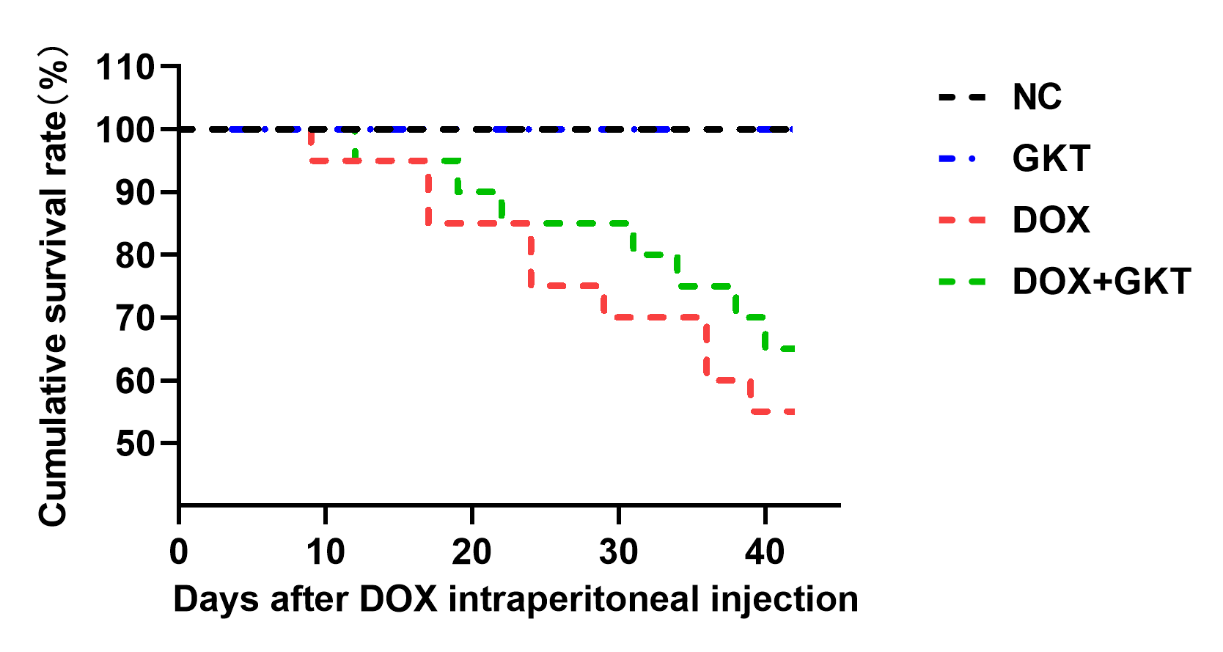


**Supplementary Figure 1. Cumulative survival rate of mice in control , GKT137831, DOX and DOX + GKT137831 groups.**

Kaplan-Meier survival analysis showed that DOX-treated group (n=20) had a significant survival decrease compared to the control group (n=10, *p* < 0.05), DOX + GKT137831 group (n=20) had no significant difference compared to DOX-treated group (*p* > 0.05). Abbreviations: DOX, doxorubicin; GKT, GKT137831.


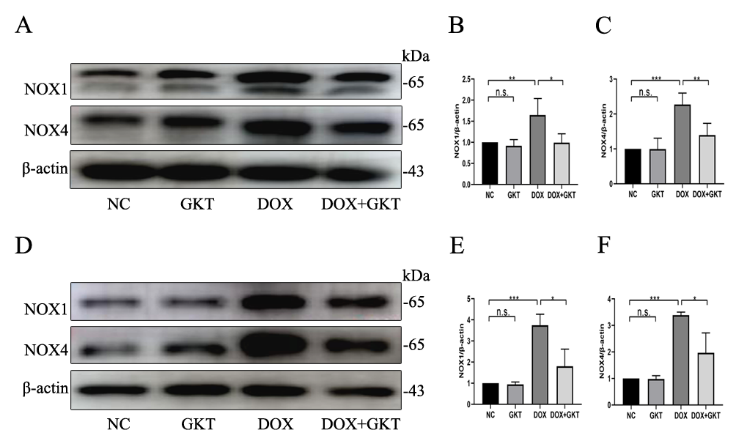


**Supplementary Figure 2. GKT137831 inhibited the protein** **expression of NOX1 and NOX4 both *in vivo* and *in vitro*.**

**(A)** Representative western blot analysis of NOX1 and NOX4 in myocardial tissue. **(B, C)** Quantification of NOX1 and NOX4 expression relative to the β-actin level (n=5 per group). **(D)** Representative western blot analysis of NOX1 and NOX4 of NRCMs. **(E, F)** Quantification of NOX1 and NOX4 expression relative to the β-actin level (n=3). * *p* < 0.05, ** *p* < 0.01, *** *p* < 0.001, n.s., not significant. Abbreviations: NOX, NADPH oxidase; DOX, doxorubicin; GKT, GKT137831.
